# Developmental Trajectories in Diagnostic Reasoning: Understanding Data Are Confounded Develops Independently of Choosing Informative Interventions to Resolve Confounded Data

**DOI:** 10.3389/fpsyg.2022.800226

**Published:** 2022-02-15

**Authors:** April Moeller, Beate Sodian, David M. Sobel

**Affiliations:** ^1^Department of Psychology, Ludwig Maximilian University of Munich, Munich, Germany; ^2^Department of Cognitive, Linguistic, and Psychological Sciences, Brown University, Providence, RI, United States

**Keywords:** diagnostic reasoning, scientific thinking, control of variables strategy (CVS), causal reasoning, experimentation, preschoolers

## Abstract

Two facets of diagnostic reasoning related to scientific thinking are recognizing the difference between confounded and unconfounded evidence and selecting appropriate interventions that could provide learners the evidence necessary to make an appropriate causal conclusion (i.e., the control-of-variables strategy). The present study investigates both these abilities in 3- to 6-year-old children (*N* = 57). We found both competence and developmental progress in the capacity to recognize that evidence is confounded. Similarly, children performed above chance in some tasks testing for the selection of a controlled test of a hypothesis. However, these capacities were unrelated, suggesting that preschoolers’ nascent understanding of the control-of-variables strategy may not be driven by a metacognitive understanding that confounded evidence does not support a unique causal conclusion, and requires further investigation.

## Introduction

There is now convergent evidence that children have sophisticated and intuitive causal reasoning abilities. Infants and preschoolers register conditional independence among events (e.g., Gopnik et al., 2001; Sobel and Kirkham, 2006), infer hidden causes from patterns of covariance (e.g., Oakes and Cohen, 1990; Saxe et al., 2005), and reason diagnostically from outcomes to likely causes (e.g., Bonawitz et al., 2012; Fernbach et al., 2012; Kimura and Gopnik, 2019). Young children’s explanations reflect their causal knowledge (e.g., Schult and Wellman, 1997) and they use explanations to facilitate their causal learning (e.g., Legare, 2012; Legare and Lombrozo, 2014; Walker et al., 2017). Taken together, these, and many other lines of investigation suggest the possibility that cognitive development in certain domains is described by a process in which children revise theories based on observation and interaction with the world (e.g., Gopnik and Meltzoff, 1997; Gopnik and Wellman, 2012). On this view, children are often described as little scientists.

But scientific thinking^1^ is not the same as causal reasoning. Scientific thinking involves not only the capacity for causal reasoning, but also a more metacognitive understanding of the relation between theory and evidence (e.g., Kuhn, 2011). This includes knowing when the data one observes are confounded [i.e., they result from a comparison in which the variable of interest as well as one or more other variables were varied (Chen and Klahr, 1999) making it impossible to determine the effect of individual variables], and designing interventions – actions on the world – to make appropriate causal conclusions. The studies that document children’s sophisticated causal reasoning often ask children to observe and interpret evidence made available to them by researchers and use that evidence to construct and revise their causal beliefs. This is not necessarily indicative of scientific thinking. To consider children’s capacities for scientific thinking, we need to examine whether they explicitly understand that the data they observe are confounded. Moreover, we need to examine whether children can generate novel information to resolve ambiguity when they observe confounded data. While these diagnostic reasoning abilities are not the only facets of scientific thinking, understanding both are required to begin to appreciate the many ways causal reasoning and scientific thinking are connected.

### Can Young Children Articulate the Difference Between Confounded and Unconfounded Evidence?

Some investigations have suggested that children do understand the difference between confounded and unconfounded data. Preschoolers selectively explore a causal system differently when shown confounded vs. unconfounded evidence (Schulz and Bonawitz, 2007). Children at this age also spontaneously demonstrate information-seeking behaviors when they observed confounded evidence (Cook et al., 2011), and they can intervene on causal systems to gain information (Gweon and Schulz, 2008). While the capacity to gain the most information about a causal structure from one’s own exploration develops into the adolescent years (e.g., Nussenbaum et al., 2020), even young children show some capacity to gain some information from their exploration. These results suggest that young children recognize the difference between confounded and unconfounded information by acting on the world in different ways.

However, caution must be taken when coming to this conclusion. Although young children can articulate the appropriate causal conclusions from observing first confounded, and then unconfounded evidence (e.g., Sobel et al., 2004), few studies show that children explicitly understand that the data they observe is confounded when they observe such data. As an example, Köksal et al. (2021) presented 5- and 6-year-olds with two blocks that together activated a machine, and asked children whether they knew that one of those two blocks on their own was efficacious. Half of their sample articulated that they could not tell from these data. This finding is consistent with research showing that a metacognitive understanding of one’s own ignorance in the case of partial information develops only around the age of 5 to 6 years (e.g., Rohwer et al., 2012).

One goal of the present investigation is to examine whether younger children can make a similar inference and trace its developmental trajectory. Moreover, we wish to examine whether there is a relation between recognizing that confounded data does not license a causal inference and being able to choose interventions that would provide the learner with potentially unconfounded evidence. We turn to this discussion in the next section.

### Can Children Generate Novel Interventions to Observe Potentially Unconfounded Data?

Understanding that evidence can be a source of causal knowledge is critical to testing and revising hypotheses (e.g., Morris et al., 2012). When does this capacity develop? When presented with a simple hypothesis about a determinate state of affairs, most 6-year-olds and almost all 8-year-olds preferred a conclusive over an inconclusive test and could justify their choice in terms of the appropriateness of the test design (Sodian et al., 1991).

But testing hypotheses about causal relations among a set of variables requires a more sophisticated strategy; reasoners must isolate variables and test their effects individually. Across three different experiments, Lapidow and Walker (2020) showed that 4- to 6-year-olds selected interventions that would provide them with the causal information necessary to disambiguate data. For example, in one of their experiments (Experiment 2), children were told that two interlocking gears would spin together on a gear toy if both were “working” or if one working gear pushed a “broken” gear; two broken gears, however, would simply be inert on the machine. They showed children that two new interlocking gears spun together when the machine was turned on and wanted to know whether both were working gears or specifically one gear (gear A) was working and the other (gear B) was broken. Children were asked whether they wanted to see gear A on the machine alone (a confounded intervention) or gear B on the machine alone (an unconfounded intervention). The majority of children chose the unconfounded intervention, and those that did were more likely to come to the appropriate causal conclusion about the efficacy of the individual gears from the data they subsequently observed. These findings reveal reasoning abilities in preschool children that could support early scientific thinking.

But, to test hypotheses about cause-effect relations among multiple variables, reasoners must go further than isolating individual variables to observe their effects – they must manipulate the variable in question while keeping all other variables constant (Tschirgi, 1980). Keeping non-focal variables constant results in an unconfounded experiment from which valid causal inferences can be made. Although early elementary school-aged children can learn this *Control of Variables* strategy via direct instruction (Chen and Klahr, 1999), they do not use this strategy spontaneously until approximately the fourth grade (Schwichow et al., 2016). There are cases in which middle school students struggle with constructing these kinds of well-controlled interventions (e.g., Schauble, 1990; Kuhn and Dean, 2005), and cases that show adult participants are not fully rational when engaging in causal learning from their own actions (Schauble, 1996; Coenen et al., 2015).

Consistent with these findings (McCormack et al., 2015; see also Meng et al., 2018) suggested that young children do not always design informative interventions in their exploration. They showed 5- to 9-year-olds a causal system and confounded evidence that could indicate multiple causal structures. They then allowed children to intervene on these systems. The oldest children in their sample were the most likely to infer the causal structure. Younger children in this study did not always design unconfounded interventions in their actions, but rather often intervened on “root” nodes – the event that caused the most outcomes.

Although young children might not be able to produce controlled experiments, most children of this age can choose which of two experiments is unconfounded (Bullock and Ziegler, 1999; Lapidow and Walker, 2020). Moreover, when provided with scaffolding and direct instruction, even preschoolers can produce controlled experiments when shown confounded data (van der Graaf et al., 2015; van Schijndel et al., 2015). Between the ages of 3 and 6, children used systematic testing strategies, rather than random actions, to choose unconfounded over confounded evidence when constructing counterevidence against a false claim (Köksal-Tuncer and Sodian, 2018). These data suggest the possibility that young children can choose informative interventions when those interventions are presented to them. The present study was designed to provide systematic evidence on the choice of informative over uninformative interventions in two control-of-variables tasks in children between the ages of 3 and 6.

### Overview of the Present Experiment

While the findings reviewed in the previous sections suggest that children have some capacity for the diagnostic inference necessary for scientific thinking before they enter formal schooling environments, there are two open questions, which motivate the current investigation. First, most studies that examine children’s ability to produce controlled experiments have not assessed whether children specifically use the control of variables strategy without prompting, as well as their metacognitive understanding of using that strategy. Second, it is not clear whether young children recognize confounded data as confounded, and the role that this capacity plays in their use of the control of variables strategy.

As a first step toward addressing such questions, we investigated 3- to 6-year-olds’ capacity to recognize that confounded data were confounded and did not support a causal conclusion on their own, and relatedly, whether the same children could choose an intervention that would provide them with informative data to disambiguate the causal structure following the control of variables strategy. We introduced children to a machine that activated when certain objects were placed on it, controlled by the experimenter. This knowledge-lean measure was used to ensure that all children had little relevant prior knowledge about the underlying causal structures they observed.

On the Interpretation of Confounded Evidence (ICE) trials, children observed that a set of objects (Duplo bricks stuck together) activated the machine. We asked whether children recognized that the evidence they observed was insufficient to draw a causal conclusion about which bricks could make the machine go and which could not, as well as to justify their response. This measure considered whether children were aware that they could not draw a causal conclusion when presented with confounded evidence.

On the Control of Variables Strategy (CVS) trials, we showed children a new set of bricks that activated the machine, and then told them that we wanted to find out if a specific brick in the set was efficacious. We asked the children to choose an intervention that would allow them to determine whether that brick had efficacy. We offered children a forced-choice in which they could manipulate one variable at a time, which could produce unconfounded data or multiple variables at a time, which was guaranteed to produce confounded data. After children made their choice, we asked them to justify their response.

Tasks used to investigate whether children have this diagnostic reasoning capacity commonly present children with problems that contain at least three variables, but the simplest cases involve only two variables and requires the understanding that only one of those variables, and not both, should be manipulated. We presented children with two types of CVS trials; in some there were only two potential causes, while in others there were three. This allowed us to vary the difficulty of this inference.

On both ICE and CVS trials, we considered whether children made the appropriate inference, but also how they justified their response. These justifications are critical because they represent more of an explicit understanding that evidence is confounded than merely saying it is, as well as more of an explicit understanding of why one would choose a particular intervention to resolve confounded information as opposed to simply making that choice. Moreover, we considered the extent to which the ability to recognize that confounded evidence is so relates to the ability to select interventions that can disambiguate such confounded evidence (i.e., whether there are relations between the ICE and CVS measures).

## Materials and Methods

### Participants

The final sample consisted of 57 children (*M*_age_ = 65.12 months, *SD* = 9.24 months; range: 41–81 months; 29 girls and 28 boys). Five additional children were tested but excluded due to color vision deficits (1) or experimenter error (4). All participants were typically developing children recruited from an urban area. Parental informed consent and child assent were obtained for all children. Sample size was determined through power analysis based on a linear regression assuming a fixed model with α = 0.05, β = 0.20 and a medium-to-large effect size (*f*^2^ = 0.25) based on Cohen (1992).

### Materials

**FIGURE 1:**
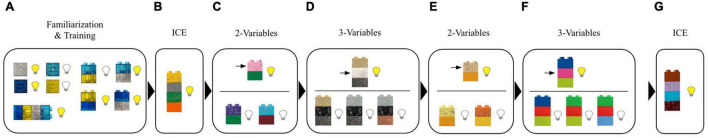
Materials and an example of the testing procedure. Yellow (dark gray) lightbulb indicates the box lit up when the corresponding object was placed on it. White (light gray) lightbulb indicates the box did not light up when the corresponding object was placed on it. **(A)** Shows the bricks used in the familiarization and training, first individually and then in combination as shown. **(B,G)** Show the objects used in the ICE trials. In **(C–F)**, the initial stick is shown above the horizontal line, with the (X) focal brick indicated by an arrow. Below the horizontal line are the corresponding test choices that children could select. See Procedure for further explanation.

The lightbox machine (based on the blicket detector, Gopnik and Sobel, 2000) was a custom-built wooden box (30 × 20 × 15 cm) with an LED strip around the top that was controlled by the experimenter via foot pedal. Forty-eight Lego Duplo bricks in 30 unique colors and patterns were used to activate the machine (see Figures 1A–G). Seven sets of bricks were used: four bricks for familiarization and training (Figure 1A), two sticks with four bricks each for the ICE trials (Figures 1B,G), two sets of three sticks with two bricks each for the two-variable CVS trials (Figures 1C,E), and two sets of four sticks with three bricks each for the three-variable CVS trials (Figures 1D,F). The bricks in all sticks were glued together so that they could not be separated.

A cardboard tray (16 × 21 cm) was used to present children with test choices. A clear plexiglass cover (17 × 24 × 8 cm) was placed over the choices to prevent children from grabbing for the options before hearing the critical questions. Eight testing versions were created to counterbalance the task materials, location of the correct choices (Left, Middle, or Right), and order of the tasks (two- or three-variable task first). We will use the order in Figure 1 to illustrate the procedure.

As part of the warm-up, children played a puzzle matching game which required them to match the mother animal with the appropriate baby animals (e.g., a chicken with her chicks). Children also performed a color vision test, based on Ishihara’s dotted circles, in which they had to trace a line with their finger.

### Procedure

Children were tested at their school setting in a separate, quiet room. Children’s session was video recorded for subsequent coding. The session lasted approximately 15 min. Children sat at a table with a female experimenter who first administered a matching puzzle game to familiarize children with the testing environment and a color vision test to ensure that children could discern among the colors used in the procedure. One child failed the color vision test and was not included in analyses.

The experimenter then introduced the lightbox and showed children that some bricks made the box light up and some did not. The experimenter told children that “tomas” (a novel label) made the box light up and bricks that that did not activate the box were “not tomas.” Children first observed two individual bricks activate the box (and labeled “tomas,” e.g., “This dark blue brick is a toma.”) and two individual bricks not activate the box (and labeled “not tomas,” e.g., “this blue sparkly brick is not a toma.”). Children were also shown five combinations of bricks (two tomas, two not-tomas, two sets of a toma and a not-toma, and all four bricks together). The combinations of bricks made the box light up as long as one of those bricks was a toma (Figure 1A). The purpose of this initial familiarization with different bricks was to show children that some bricks are causal, and some bricks are inert and that the box had a disjunctive structure. Consequently, children should know that novel bricks could be causal or inert and that combinations of bricks could include only causal bricks, only inert bricks, or a combination of both. This introduction took approximately 5 min and was followed by a memory check for which bricks made the box light up. If children failed the memory check, the initial training with individual bricks was repeated.

#### Interpretation of Confounded Evidence (ICE) Trials

Children observed that a stick of four bricks, placed horizontally on the box, activated the box (Figure 1B). Children were asked, “Can you know for sure which of the bricks are tomas or can you not know for sure?” Children who indicated they knew which bricks were causal were asked if they were certain or not and to explain how they knew. Children who indicated they could not know which bricks were causal were asked to explain why they could not know. In both cases, the explanations provide us with information as to whether and how children understood the inference they made.

#### Control of Variables Trials

Children received two types of CVS trials. In the two-variable CVS trials, the experimenter placed a stick of two bricks (X and Y) on the light box, which activated (Figure 1C). The experimenter pointed to the top brick and said, “We want to find out if this brick (X) is a toma.” The XY stick was placed in front of the child. Two additional sticks were then placed on the table and the experimenter explained: “You can pick one of these sticks to place on the box to find out if the X brick is a toma. Which stick is the best to find out if the X brick is a toma?” One stick (the unconfounded choice) swapped the X brick with a novel color (Z) but kept the Y brick (so the stick was Z and Y). The other stick (the confounded choice) swapped both bricks, resulting in a stick of two novel colors (P and Q). After children indicated their choice, the experimenter asked, “Why do you think this stick is the best to find out if the X brick is a toma?”

Finally, the experimenter placed the chosen stick horizontally on the box. The box did not light up. Children were asked to interpret the evidence generated by their experiment: “Now do you know if the X brick is a toma, not a toma, or do you not know?” They were also asked if they were certain or not and to provide an explanation for their answer.

The procedure for the three-variable CVS trial was the same as for the two-variable trial, with two exceptions. First, children were shown that a stick of three bricks made the box light up and were asked to find out if the middle brick (X) was a toma (Figure 1D). Second, instead of only two choices, children were given three sticks as choices. The unconfounded choice varied the brick in question (changed X to Z) but kept the other two bricks the same; the other two choices were both confounded: A second stick varied the brick in question as well as an additional brick; the third stick varied all three bricks, resulting in a stick with three novel colors. With these changes, we increased the level of difficulty of the task.

Children performed two trials of each task type (Figures 1E,F) and then finished with a second ICE task (Figure 1G). The order of the CVS tasks was counterbalanced, such that half of the participants received a two-variable CVS task following the first ICE task and half received a three-variable CVS task following the first ICE task. The number of variables in the CVS tasks then alternated. With this procedure, we could observe both children’s initial response to each task and the consistency of their responding across two trials for a more robust measure of their capabilities. We could also examine the potential influence of the ICE trial on CVS and vice versa.

### Coding

#### Interpretation of Confounded Evidence Trials

Responses that indicated children did not know which bricks were tomas were coded as correct. This category includes children who claimed they could not know in the first question (“Can you know for sure which of the bricks are tomas or can you not know for sure?”) as well as children who first claimed they did know, but then indicated they were not sure. The second category was an incorrect claim of knowledge and included responses that indicated children knew which bricks were tomas and were certain.

Next, we coded explanations for how children knew or why they could not know which bricks were tomas. All explanations for how they knew were coded as incorrect. Explanations for why they could not know were coded as correct or incorrect. A correct explanation indicated that the bricks were stuck together and could not be isolated and tested individually (see Table 1 for examples). We further considered the proportion of trials on which children generated a correct knowledge claim and a correct justification. We refer to this pattern of performance as generating a *Robust* response.

**TABLE 1:** Examples of explanations for the ICE trials.

Correct	Incorrect
I can’t know because:	I don’t know
The bricks are stuck together	I can’t know because:
I can’t try them out	These are different bricks
It could be any of the bricks	I haven’t seen these bricks before
I haven’t seen which ones light up	No one told me
I can’t try them one at a time	I know because:
	It’s yellow like the sun;
	They sparkle; they are pretty
	They made the box light up
	My mom told me; I have a book about them
	I’m a big kid; I think so

#### Control of Variables Trials

In both the two-variable and three-variable CVS trials, we first coded whether children selected the correct intervention. This involved choosing the unconfounded choice as opposed to a confounded choice on each trial, as that response manipulates the focal variable (substituted Z for X) while keeping all other variables constant.

We next considered justifications for why children made that choice. Relevant justifications referred to the absence of the X brick in the choice, the presence of one or both of the control bricks in the choice, or both the absence of the X brick and the presence of the control brick(s) (see Table 2 for examples). Other justifications, such as color preference, were coded as irrelevant. Additionally, we defined children as generating a *Robust* response if they generated a relevant justification for a correct intervention choice.

**TABLE 2:** Examples of children’s justifications for their test selection.

Relevant justifications
This brick is different from the X brick; This stick doesn’t have the X brick
This stick also has this (control) color
This brick is the same as that brick (control) and this brick is the same as that brick (control)
These two bricks are the same as those two bricks (controls)
These sticks are the same, but it doesn’t have this (X) brick; Only this brick (X) is different

**Irrelevant justifications**

I don’t know; It just is; My mom told me; I have a book about it
I like this one; This one is pretty; These look nice together
I picked the other one last time; Let’s try it; We haven’t tried it yet
It is a lighter; Maybe it lights up; It is not a lighter
Because it is similar to the test stick

At the end of each CVS task, children were asked to interpret the evidence generated by their choice. If children chose the correct stick to test, then we considered whether they claimed that they were certain that the X brick was a toma. If they chose an incorrect stick to test, then we considered whether they could not be sure that the X brick was a toma.

#### Reliability

All videos were coded by the first author and an independent rater. In the ICE task, agreement was 93% (Kappa = 0.85) for Knowledge Claims and 97% (Kappa = 0.87) for justifications. In the CVS tasks, agreement was 98% (Kappa = 0.96) for Choices and 93% (Kappa = 0.72) for justifications. Disagreements were resolved by a discussion of the two raters.

## Results

Table 3 shows performance on the ICE and two CVS trials. Table 4 shows the results of all Generalized Estimating Equation models. We first consider performance on these trials individually, and then look at the relations among the tasks.

**TABLE 3:** Performance across two trials of the ICE and CVS tasks.

			Percentage of responses (out of 2)		
Task	Sub-task	Response	2	1	0
ICE		Correct knowledge claim	23% (13)	32% (18)	45% (26)
		Robust	16% (9)	12% (7)	72% (41)
CVS	Two-variable	Correct choice	46% (26)	40% (23)	14% (8)
		Robust	16% (9)	28% (16)	56% (32)
		Correct interpretation	23% (13)	47% (27)	30% (17)
		Robust interpretation	37% (21)	40% (23)	23% (13)
	Three-variable	Correct choice	17% (10)	51% (29)	32% (18)
		Robust	7% (4)	30% (17)	63% (36)
		Correct interpretation	16% (9)	58% (33)	26% (15)
		Robust interpretation	25% (14)	56% (32)	19% (11)

*Correct knowledge claim refers to children’s spontaneous claim of their lack of knowledge (i.e., that they cannot know which bricks make the box light up on the ICE Trials). Correct choice refers to children’s selection of an intervention on the CVS Trials. Responses were considered robust when children provided a correct or relevant verbal explanation in addition to a correct response or choice.*

**TABLE 4:** Generalized estimating equation model results.

Predictor	B	SE	Wald	df	*p*	95% CI	
						Lower	Upper
**ICE**							
Age	0.09	0.03	8.10	1	0.004	0.03	0.14
Trial	0.67	0.35	3.60	1	0.058	−0.02	1.36
**ICE robust**							
Age	0.11	0.03	12.83	1	<0.001	0.05	0.16
Trial	0.59	0.30	3.87	1	0.049	0.002	1.18
**CVS choice**							
Age	−0.01	0.01	0.14	1	0.712	−0.03	0.02
Task	0.95	0.27	12.61	1	<0.001	0.42	1.47
Trial	0.38	0.27	1.92	1	0.166	−0.16	0.91
**CVS robust**							
Age	0.06	0.03	5.84	1	0.016	0.01	0.11
Task	0.19	0.24	0.63	1	0.428	−0.28	0.65
Trial	0.28	0.24	1.38	1	0.240	−0.19	0.75
**CVS interpretation**							
Age	0.01	0.01	0.28	1	0.599	−0.02	0.03
Task	0.004	0.23	<0.00	1	0.988	−0.46	0.46
Trial	0.26	0.30	0.73	1	0.394	−0.34	0.86
CVS choice	−0.30	0.33	0.80	1	0.370	−0.95	0.35
**CVS interpretation**							
Age	−0.002	0.01	0.02	1	0.891	−0.03	0.03
Task	0.05	0.23	0.04	1	0.845	−0.41	0.50
Trial	0.25	0.31	0.64	1	0.423	−0.36	0.86
CVS robust	−0.78	0.35	4.84	1	0.028	−1.47	−0.84

### Interpretation of Confounded Evidence Trials

For the ICE trials, the dependent measure was whether children responded in a way that indicated an understanding of the inconclusiveness of evidence by answering that they did not know which bricks were tomas. Overall, children generated this response on 39% of the trials. As a preliminary analysis, we built a Generalized Estimating Equation (GEE) with an independent working correlation matrix, a binomial distribution, and a cumulative logit link function (Zeger and Liang, 1986; Zeger et al., 1988) looking at the role of gender and task materials on children’s knowledge claim responses. Both of these factors were not significant (both *p*-values > 0.34), so we will not consider them further.

For the main analysis we constructed a new GEE looking at the role of age and trial (first vs. second). This model revealed a main effect of age, *B* = 0.09, *SE* = 0.03, [95% CI = 0.03, 0.14], Wald χ*^2^*(1) = 8.10, *p* = 0.004, but no significant effect of trial, *B* = 0.67, *SE* = 0.35, [95% CI = −0.02, 1.36], Wald χ*^2^*(1) = 3.60, *p* = 0.06.

We conducted a similar GEE on Robust responding, which children generated on 22% of the trials. Again, we considered the role of age and trial. This model also revealed a main effect of age, *B* = 0.11, *SE* = 0.03, [95% CI = 0.05, 0.16], Wald χ*^2^*(1) = 12.83, *p* < 0.001. As children developed between the ages of 3 and 6, they were more likely to claim that confounded evidence was confounded and justify the reason for their inference appropriately. There was also an effect of trial, *B* = 0.59, *SE* = 0.30, [95% CI = 0.002, 1.18], Wald χ*^2^*(1) = 3.87, *p* = 0.05. Children were more likely make a robust response on the first ICE trial (26%) than the second (18%).

### Control of Variables Trials

For the CVS trials, we first considered whether children chose the response that indicated a controlled experiment as the dependent variable (shown in Table 3). Children made this response on 66% of the trials in the two-variable case and 43% of the trials in the three-variable case. As a preliminary analysis, we built a GEE looking at the role of gender, the order in which children received the tasks, the task materials, and the location of the correct choice on children’s selection. None of these factors were significant (all *p*-values > 0.27), and we will not consider them further.

For our main analysis, we constructed a GEE examining whether children chose the response that indicated a controlled experiment on the CVS tasks, looking at the role of age, task (two-variable vs. three-variable), and trial (first vs. second). This model revealed only a main effect of task, with performance better on the two-variable CVS trial than the three-variable CVS trial, *B* = 0.95, *SE* = 0.27, [95% CI = 0.42, 1.47], Wald χ*^2^*(1) = 12.61, *p* < 0.001. There were no significant effects for age, *B* = −0.01, *SE* = 0.01, [95% CI = −0.03, 0.02], Wald χ*^2^*(1) = 0.14, *p* = 0.712, or trial, *B* = 0.38, *SE* = 0.27, [95% CI = −0.16, 0.91], Wald χ*^2^*(1) = 1.92, *p* = 0.17. Of course, the two-variable trial only had two response outcomes, whereas the three-variable trial had three, so we also considered performance compared to chance responding. Performance across two trials on the two-variable CVS tasks was different than expected by chance, χ^2^(2) = 13.49, *p* = 0.001, Cohen’s *w* = 0.49; performance across two trials on the three-variable tasks was marginally different from chance, χ^2^(2) = 4.78, *p* = 0.09, Cohen’s *w* = 0.29.

We next considered the justifications children generated for responses to the CVS trials. Across the trials, 38% of all justifications were coded as relevant and children generated robust responses on 30% of the two-variable CVS trials and 22% of the three-variable CVS trials. We constructed a similar GEE to examine children’s robust CVS performance, looking at the role of age, task, and trial. The model revealed only a main effect of age, *B* = 0.06, *SE* = 0.03, [95% CI = 0.01, 0.11], Wald χ*^2^*(1) = 5.84, *p* = 0.02. There were no significant effects for task, *B* = 0.19, *SE* = 0.24, [95% CI = −0.28, 0.65], Wald χ*^2^*(1) = 0.63, *p* = 0.43, or trial, *B* = 0.28, *SE* = 0.24, [95% CI = −0.19, 0.75], Wald χ*^2^*(1) = 1.38, *p* = 0.24. Older children in our sample were more likely than younger children to provide a robust response on the CVS trials.

Next, we focused on children’s interpretation of the evidence generated by their choice in the CVS trials. After children chose which stick to place on the machine, they observed the results of their choice, critically, for both choices, the stick did not activate the machine. If children chose the unconfounded stick (i.e., the correct choice), they can now conclude that the X brick was a toma, whereas if children chose a confounded stick, they should still be uncertain in their conclusion. In response to this question, children could state that they knew the X brick was a toma, was not a toma, or that they did not know.

We constructed two GEE models examining whether children stated that they knew the X brick was a toma. The first looked at the role of age, task, trial, and choice (unconfounded or confounded intervention). None of these factors were significant (all *p*-values > 0.37). Children’s interpretation of the outcome of their experiment was not affected by whether they had previously chosen a controlled test. Both children who chose a controlled test and children who chose a confounded test were equally likely to claim that the X brick was a toma.

The second replaced children’s initial choice with whether children generated a robust response (i.e., chose the unconfounded and generated a relevant justification for that choice). This model revealed a main effect of robust performance on the CVS tasks, *B* = 0.78, *SE* = 0.35, [95% CI = 0.08, 1.47], Wald χ*^2^*(1) = 4.84, *p* = 0.03. There were no significant effects for age, *B* = 0.002, *SE* = 0.01, [95% CI = −0.03, 0.03], Wald χ*^2^*(1) = 0.02, *p* = 0.89, task, *B* = −0.05, *SE* = 0.23, [95% CI = −0.50, 0.41], Wald χ*^2^*(1) = 0.04, *p* = 0.85, or trial, *B* = −0.25, *SE* = 0.31, [95% CI = −0.86, 0.36], Wald χ*^2^*(1) = 0.64, *p* = 0.42. Robust performance on a CVS task (i.e., making the correct choice and justifying it appropriately) uniquely predicted making a correct interpretation of the experiment outcome rather than an incorrect interpretation.

#### Relations Between the Interpretation of Confounded Evidence and Control of Variables Strategy Trials

Our final set of analyses considered the relation between performance on the ICE and CVS trials. Table 5 shows bivariate correlations among our dependent measures as well as age (in months).

**TABLE 5:** Bivariate correlations between the ICE and CVS tasks.

Variables	1	2	3	4	5	6	7
Age (in months)	–						
ICE Knowledge Claim	0.39**	–					
ICE Robust	0.37**	0.76***	–				
2-Choice CVS	−0.27*	–0.15	–0.10	–			
2-Choice Robust CVS	0.20	0.02	–0.09	0.48***	–		
3-Choice CVS	0.21	0.20	0.19	0.09	0.30*	–	
3-Choice Robust CVS	0.36**	0.13	0.04	0.24†	0.50***	0.64***	–

^†^p < 0.10, *p < 0.05, **p < 0.01, ***p < 0.001.

There are three findings of note here. First, note that age significantly correlates with performance on the ICE trials, but not reliably with performance on the CVS trials (indeed, choices on the two-variable CVS trials were negatively correlated with age). Robust performance was positively correlated with age on the three-variable trials, but not on the two-variable trials.

Second, choosing the unconfounded choice on the two-variable and three-variable trials did not significantly correlate with one another, *r*(55) = 0.09, *p* = 0.50. However, generating robust responses on the two-variable and three-variable trials were significantly correlated, *r*(55) = 0.50, *p* < 0.001, and this correlation held controlling for age, *r*(55) = 0.47, *p* < 0.001. This suggests that the choices children make when asked to select which intervention to conduct might be independent of their understanding of why they might be making that choice. Their understanding of why they are making those choices might be related to one another.

Third, there are no significant bivariate correlations between the ICE trials and the CVS trials, including between robust performance on the ICE and CVS trials. This suggests that performance on the ICE and CVS trials were independent of one another. To investigate this further, we constructed the same GEE models to analyze the CVS trials as before but added whether children generated a robust performance on the first ICE trial (as they always were given that trial before the CVS trials and because performance potentially differed between the two ICE trials – children who respond correctly on the first trial potentially show a better understanding of ICE than overall ICE performance). This variable did not significantly predict performance on the CVS trial (both *p*-values > 0.21). The addition of the first ICE trial resulted in a worse goodness of fit value for the model (indicated by QIC score), suggesting that adding it to the model did not allow us to better explain performance on the CVS trials. Finally, performance on the CVS tasks did not predict performance on the second ICE trial (all *p*-values > 0.05). This suggests two conclusions. First, robust performance on the ICE and CVS tasks are independent from one another, and robust performance is not simply the result of children’s more sophisticated ability to articulate the justifications of their choices. Second, only a robust understanding of the CVS tasks was guided by a task-independent understanding of the CVS strategy.

## Discussion

The present study measured 3- to 6-year-olds’ ability to articulate whether data they observed were confounded regarding a particular causal structure (the ICE trials) and whether they could choose interventions that followed the Control of Variables strategy in order to learn about a novel causal system (the two types of CVS trials). Looking at performance on these measures allows us to consider the development of both facets of causal reasoning, but also allowed us to consider whether performance on these of tasks were related to one another.

In the ICE measures, just over half of the children in the sample recognized the ambiguity of the presented data in at least one trial, with around a quarter of the children able to articulate that confounded evidence did not support a particular conclusion. In the two-variable CVS task, a majority of the children (∼70%) could recognize a controlled test with two variables on at least one trial and showed above-chance performance overall. Just under half of the children in the sample were also able to justify why they chose the intervention that followed the Control of Variables strategy in a rational way on at least one trial. Similarly, in the three-variable CVS task more than two-thirds of the children could recognize a controlled test with three variables at least once, with over one third of the children able to justify their intervention.

Overall, as children in the sample got older, they were more likely to respond accurately on the ICE trial as well as generate a relevant explanation for why the ambiguous data they observed were confounded. The relation with age was more complex on the CVS trials, as younger children were more likely to respond accurately on the two-variable CVS trial (with no significant relation to robust responding), while older children were more likely to show robust responses on the three-variable CVS trial (with no significant relation to just their choice on this trial type). Moreover, performance on the ICE trials and performance on the CVS were also unrelated to one another, both in terms of the responses that children generated, but also whether their justification was relevant and reflected metacognitive understanding. Performance on the ICE measure did not predict a significant amount of variance on responses to the CVS trials, nor did performance on the CVS tasks predict children’s performance on the second ICE trial. Performance on the two types of CVS trials were also not related to one another.

These findings also allow us to address two potential concerns with the study. First, the answer we counted as “correct” on the CVS trials is the one that is more perceptually similar to the demonstrated item, so children might have responded on the basis of that similarity. While it is unclear to us why children might be more likely to use perceptual similarity vs. perceptual dissimilarity as a basis for response, the difference in performance between the two types of CVS trials suggests that children were not simply informed by this response bias. If children had simply used perceptual similarity to select the bricks to use, they would be equally correct across the trials. Moreover, choices on the two types of CVS trials did not correlate with one another, but robust performance did. This suggests that a simple perceptual bias affecting only children’s choices did not underlie the present finding.

Second, as mentioned above, it is possible that robust responses reflect children’s increased linguistic capacity to articulate a relevant, potentially more metacognitive explanation. For example, Tippenhauer et al. (2020) demonstrated that between the ages of 3 and 5, children both generate and endorse more non-circular definitions in the way they justify events. However, we do not think this is a likely explanation of the present findings. Robust responding on the ICE and the two kinds of CVS trials were not correlated with one another, and not all robust responding correlated with age. This suggests that while children’s linguistic capacities to generate relevant explanations might be a necessary part of robust understanding of ICE and CVS, it is insufficient to suggest that performance on these tasks are explained by a common mechanism of language development.

The present results show that even preschoolers can select a conclusive test of a hypothesis, consistent with several studies that have shown similar abilities in older age groups (e.g., Piekny and Maehler, 2013; Koerber and Osterhaus, 2019; Lapidow and Walker, 2020). Moreover, these results suggest that preschoolers have an early capacity for the diagnostic reasoning necessary to select interventions that produce unconfounded data, even without any explicit instruction or support, at least in controlled and knowledge-lean setting. This expands on existing studies that show young children can make similar diagnostic inferences when given such support (van der Graaf et al., 2015). Taken together, these data suggest that the preschoolers in these studies possess a nascent understanding of scientific inquiry, particularly in their ability to select interventions to learn causal relations.

That said, an interesting facet of the present data is that performance on the two-variable CVS trial negatively correlated with age – that is, the younger children in our sample were more likely to respond correctly on this trial, even though there was not a significant correlation between age and robust responding on this measure. This again points out that robust responding is unlikely to be caused by children’s advancing linguistic competence. Rather, it suggests the possibility that even performance on the two-variable and three-variable CVS trials differed. A speculative explanation is that younger children were more driven by just making a perceptually similar response. This would result in correct performance on this task without any relevant understanding of the rationale for this choice. The three-variable task offers more perceptually similar trials, making it an unlikely strategy to use, hence the absence of a correlation with age on this task, as well as the absence of a significant correlation between the two- and three-variable CVS task choices. Rather, the relation between robust responding on the two- and three-variable CVS tasks, which was significant independent of age, suggests that some children have nascent understanding of CVS in these tasks.

However, this does not mean that scientific thinking is fully developed in the preschool years. Performance on the ICE trials showed that about half of the children in the sample recognized that the data they observed was confounded. This understanding, and more importantly the ability to articulate explicitly that the data were confounded in these trials, improved with age. This suggests that even if young children might not recognize that confounded data is confounded, they may often be able to choose appropriate intervention strategies. Conversely, children who did recognize confounded evidence as uninformative may still have failed to use appropriate strategies on the CVS tasks. While it is commonly assumed that mature strategy use is driven by a metacognitive understanding of the need to distinguish between alternative possibilities in order to gain causal knowledge, early strategy use in preschoolers may be based on a partial understanding of the necessity to perform comparisons by varying one factor and keeping all others constant, rather than a metacognitive understanding that using engaging in this strategy will specifically result in appropriate acquisition of causal knowledge. Children may recognize the ambiguity of confounded evidence without necessarily inferring an appropriate strategy for disambiguation. Moreover, children might also select the intervention necessary to observe unconfounded evidence without knowing that the evidence they have seen already is confounded. Such a partial or fragmented understanding may account for some apparently contradictory findings in the literature. While children might choose an appropriately constructed intervention in a controlled setting, they might not do so naturally because they might not recognize whether data they observe is confounded.

What is common across many of these studies, including the present one, is the use of a knowledge-lean paradigm for testing children’s reasoning. Such a paradigm has advantages and disadvantages. An advantage is that eliminating the role that children’s prior knowledge might play better isolates their reasoning capacities, as opposed to their understanding of or interest in a particular context. Children may come to the laboratory with different experiences and interests in particular scientific contexts, such as floating and sinking, spring tension, or the speed of racecars, which are all commonly used ways of testing scientific thinking [and commonly used examples in early science classrooms (see e.g., NGSS Lead States, 2013)]. Studies have shown that measures of children’s scientific reasoning can be influenced by their prior beliefs or knowledge about the task content (e.g., Kuhn et al., 1988; Amsel and Brock, 1996; Weisberg et al., 2020). Measuring their reasoning independent of scientific content better describes children’s capacity for the processes that underlie scientific thinking. But this advantage can also be seen as a disadvantage if one particularly wants to apply these findings to a science classroom. An open question is whether the capacities for scientific thinking described here replicate when using contexts more often found in scientific classrooms.

We also designed the study such that the unconfounded intervention would yield the data necessary to learn the causal structure. While this can be seen as an advantage of using a knowledge-lean method – the ability to control what data children observe – it is clearly not a reflection of real-world scientific thinking. In most experiments, there is no guarantee that accurately using the control of variables strategy to manipulate a variable on any given experiment will result in obtaining the data necessary to come to the appropriate causal conclusion; one must not only use the control of variables strategy correctly, but also control the right variable.

To conclude, the present results are consistent with previous findings showing that preschoolers can select a conclusive test of a hypothesis (Piekny and Maehler, 2013; Koerber and Osterhaus, 2019) and that they have an early capacity for selecting interventions that reflect the Control of Variables Strategy (van der Graaf et al., 2015), but their capacity for recognizing that data are confounded is potentially developing into the elementary-school years. Beyond this, the results show that young children possess these abilities even without any explicit instruction or support, at least in a controlled and knowledge-lean setting. While preschoolers possess a nascent understanding of scientific thinking, performance on the present measures leaves much room for improvement. This highlights that complexity of the inferences involved in scientific thinking are important for children’s capabilities and a limitation of the present work, which is that we only tested children between the ages of 3–6 (before the sample entered formal schooling).

Finally, it would be interesting to consider how older children perform on these measures, and particularly whether performance on the ICE and CVS tasks become more related to one another in a sample of children older than considered here. As children enter formal schooling, their information processing abilities improve, as do their metacognitive abilities, which might make it easier for them to relate their understanding of what they do not know to specific implemental designs for controlled experiments. Moreover, formal learning environments introduce them to science and scientific reasoning, potentially in different ways than they have seen previously. This might also allow them to begin to understand the control of variables strategy explicitly, particularly when instructed (as suggested by Chen and Klahr, 1999), and apply it to their everyday thinking.

## Data Availability Statement

The raw data supporting the conclusions of this article will be made available by the authors, without undue reservation.

## Ethics Statement

The studies involving human participants were reviewed and approved by the Ethikkommission der Fakultät für Psychologie und Pädagogik der Ludwig-Maximilians-Universität München. Written informed consent to participate in this study was provided by the participants’ legal guardian/next of kin.

## Author Contributions

AM and BS contributed to conception and design of the study. AM collected the data, organized the database, and wrote the first draft of the manuscript. AM and DS performed the statistical analysis. AM, BS, and DS wrote sections of the manuscript. All authors contributed to manuscript revision, read, and approved the submitted version.

## Conflict of Interest

The authors declare that the research was conducted in the absence of any commercial or financial relationships that could be construed as a potential conflict of interest.

## Publisher’s Note

All claims expressed in this article are solely those of the authors and do not necessarily represent those of their affiliated organizations, or those of the publisher, the editors and the reviewers. Any product that may be evaluated in this article, or claim that may be made by its manufacturer, is not guaranteed or endorsed by the publisher.

## Footnotes

1 Critically, in this paper we want to distinguish the process of scientific *thinking* from reasoning about scientific *content*. Scientific thinking about the ambiguity of evidence or about what interventions are necessary to disambiguate causal structure involves a process of reasoning that does not necessarily have to involve scientific content. While some researchers seem to equate these terms (i.e., scientific thinking or reasoning is about scientific content, e.g., Dunbar and Fugelsang, 2005), philosophy of science often argues that the process of such reasoning is independent of the content (Hacking, 1994).
